# Dyke–Davidoff–Masson syndrome: Classical imaging findings

**DOI:** 10.4103/1817-1745.76108

**Published:** 2010

**Authors:** Paramdeep Singh, Kavita Saggar, Archana Ahluwalia

**Affiliations:** Department of Radiodiagnosis, Dayanand Medical College & Hospital, Ludhiana, Punjab, India

**Keywords:** Cerebral hemiatrophy, Dyke–Davidoff–Masson syndrome

## Abstract

A 15-year-old female presented with seizures, right-sided hemiparesis, hemiatrophy of the right side of the body and mental retardation. MRI brain revealed characteristic features diagnostic of congenital type of cerebral hemiatrophy or Dyke–Davidoff–Masson syndrome.

A 15-year-old female presented with seizures, cognitive impairment and right-sided hemiparesis since early childhood. On examination, she had hemiatrophy of the right side of the body with spastic hemiparesis and incomplete achievement of mental milestones. Magnetic resonance imaging (MRI) brain revealed atrophy of left cerebral hemisphere, cerebral peduncle, basal ganglia and thalamus. There was ipsilateral midline shift and ventricular dilatation along with skull vault thickening and prominent frontal sinus, suggestive of congenital type of cerebral hemiatrophy (CH) or Dyke–Davidoff–Masson syndrome (DDMS) [Figures 1A–C].

**Figure 1A:**
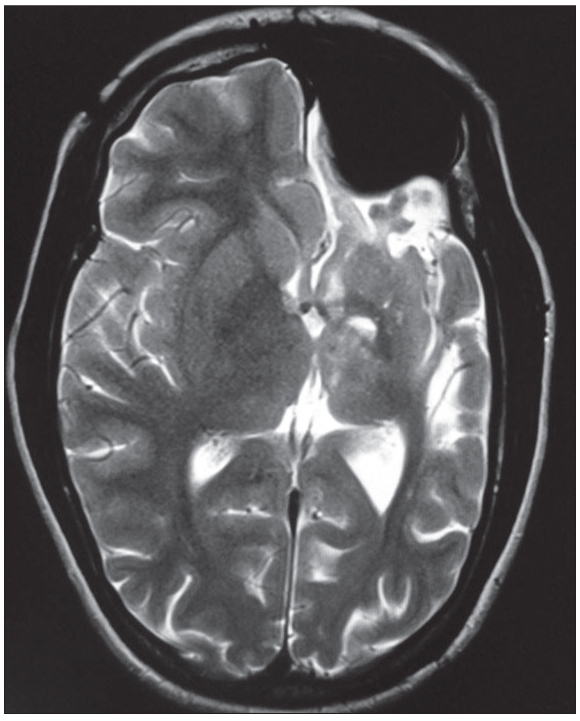
Axial T2-weighted image: Left cerebral hemiatrophy, ipsilateral occipital horn dilatation, ipsilateral midline shift, hypoplasia of thalamus, caudate nucleus, and lentiform nucleus are demonstrated. In addition, ipsilateral pneumosinus dilatans (frontal) is seen

**Figure 1B:**
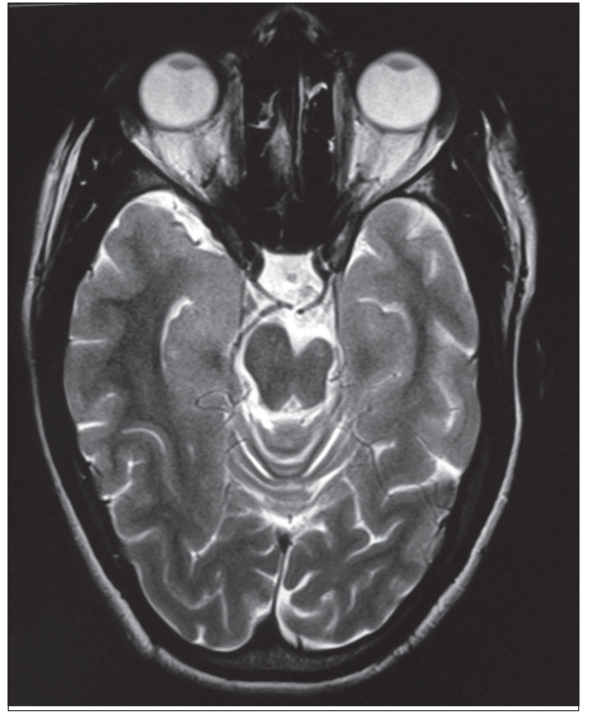
Axial T2-weighted image: Hypoplasia of left cerebral peduncle

**Figure 1C:**
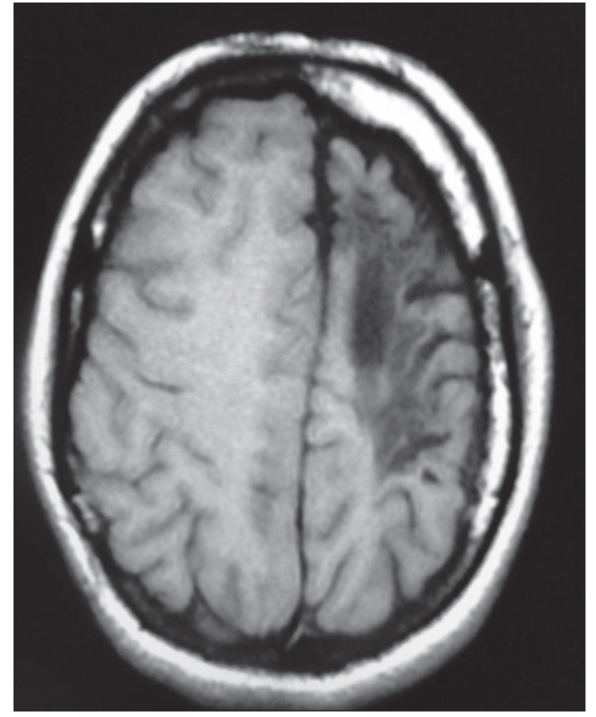
Axial T1-weighted image: Left cerebral hemiatrophy with calvarial thickening. Hypointensity in white matter represents gliosis

Clinically, patients present with seizures, facial asymmetry, contralateral hemiparesis and mental retardation. The underlying etiology is cerebral insult that may occur *in utero* or early in life. Prenatal causes include congenital anomalies, cerebral infarction, vascular malformations and infections. Perinatal causes are birth trauma, hypoxia and intracranial bleed. Postnatal hemiatrophy can develop secondary to cerebral trauma, tumors, infections and febrile seizures. Infantile (congenital) type of DDMS, in contrast to adult (acquired) DDMS, shows enlargement of calvarium, diploic space and paranasal sinuses. These compensatory cranial changes occur to take up the relative vacuum created by the atrophied cerebral hemisphere.[1,2] Shen *et al*.[3] depicted three MR imaging patterns of cerebral hemiatrophy: MR imaging pattern I corresponds to diffuse cortical and subcortical atrophy; pattern II corresponds to diffuse cortical atrophy coupled with porencephalic cysts; and pattern III corresponds to previous infarction with gliosis in the middle cerebral artery (MCA) territory. In our case, pattern III was present. The atrophied cerebral hemisphere will have prominent sulcal spaces if the vascular insult occurs after birth or after end of sulcation. However, if ischemia occurs during embryogenesis when the formation of gyri and sulci is deficient, prominent sulcal spaces will be absent.[4]

Children with medically refractive epilepsy and hemiplegia may be candidates for hemispherectomy, which is helpful in eradicating or significantly reducing seizures in 85% of patients.[5]

MRI is a valuable method of examination in the analysis of cerebral hemiatrophy as it has the ability to bring to light changes in the cerebral hemispheres as well as highlighting bony structural changes and thus differentiating between congenital and acquired types of DDMS.
